# Antibiotic Use and Bacterial Infection in COVID-19 Patients in the Second Phase of the SARS-CoV-2 Pandemic: A Scoping Review

**DOI:** 10.3390/antibiotics11080991

**Published:** 2022-07-23

**Authors:** Wenjuan Cong, Beth Stuart, Nour AIhusein, Binjuan Liu, Yunyi Tang, Hexing Wang, Yi Wang, Amit Manchundiya, Helen Lambert

**Affiliations:** 1 Department of Population Health Sciences, Bristol Medical School, University of Bristol, 39 Whatley Road, Bristol BS8 2PS, UK; nour.alhusein@bristol.ac.uk (N.A.); binjuan.liu@bristol.ac.uk (B.L.); yy.tang@bristol.ac.uk (Y.T.); ip20541@bristol.ac.uk (A.M.); h.lambert@bristol.ac.uk (H.L.); 2 Centre for Evaluation and Methods, Faculty of Medicine and Dentistry, Wolfson Institute of Population Health, Queen Mary University of London, Yvonne Carter Building, 58 Turner Street, London E1 2AB, UK; bls1@soton.ac.uk; 3 Primary Care and Population Science, University of Southampton, Aldermoor Health Centre, Aldermoor Close, Southampton SO16 5ST, UK; 4 Key Laboratory of Public Health Safety of Ministry of Education, School of Public Health, Fudan University, 130 Dongan Road, Shanghai 200243, China; wanghexing@fudan.edu.cn (H.W.); 17301020069@fudan.edu.cn (Y.W.)

**Keywords:** COVID-19 patients, antibiotic use, bacterial infection, antibiotic stewardship, frequently prescribed antibiotics, resistant pathogens

## Abstract

This scoping review aimed to explore the prevalence and patterns of global antibiotic use and bacterial infection in COVID-19 patients from studies published between June 2020 and March 2021. This review was reported in line with the Preferred Reporting of Systematic Reviews and Meta-Analyses (PRISMA) extension for Scoping Reviews, and the protocol is registered with the Open Science Framework. Compared with our previously-published review of the period (December 2019–June 2020), the antibiotic prescribing rate for COVID-19 patients (June 2020–March 2021) was found to have declined overall (82.3% vs. 39.7%), for mild and moderate patients (75.1% vs. 15.5%), and for severe and critical patients (75.3% vs. 48.3%). The seven most frequently prescribed antibiotics in COVID-19 patients were all on the “Watch” list of the WHO AWaRe antibiotics classification. The overall reported bacterial infection rate in COVID-19 patients was 10.5%, and the most frequently reported resistant pathogen in COVID-19 patients was *Staphylococcus aureus*, followed by *Pseudomonas aeruginosa*, *Escherichia coli*, and *Klebsiella pneumoniae*. There is an urgent need to establish comprehensive and consistent guidelines to assist clinicians in selecting appropriate antibiotics for COVID-19 patients when needed. The resistance data on the most frequently used antibiotics for COVID-19 patients for certain resistant pathogens should be closely monitored.

## 1. Introduction

In 2014, the O’Neill review on Antimicrobial Resistance, commissioned by the UK Government, estimated that antimicrobial resistance (AMR) could kill up to 10 million people per year by 2050 [1]. Around 1.2 million deaths in 2019 were confirmed to be attributed to AMR [2]. A study from the U.S. compared the rate of AMR infections in 271 hospitals between July 2019 and February 2020 with the rate between March and October 2020, and found the AMR rates to be similar (3.54 per 100 admissions vs. 3.47 per 100 admissions) [3]. In the first phase of the pandemic, from December 2019 to June 2020, we found the antibiotic prescribing rate for COVID-19 patients to have reached 82.3% [4]. This was in line with several other studies [5,6,7] that also documented an initial surge in antibiotic use for COVID-19 patients and widespread use of empiric antibiotic therapy in hospitalized COVID-19 patients [8,9]. At this early stage of the pandemic, COVID-19-related concerns and unknowns, difficulty in the rapid exclusion of bacterial infections and a lack of treatment guidelines pushed healthcare professionals to err on the side of treating with antibiotics, as both directed and empirical therapy [4,10]. Retrospectively, antibiotic stewardship was compromised in the treatment of COVID-19 patients in the early stage of the pandemic.

However, several studies found that antibiotic use did not prevent disease deterioration and was not associated with lower mortality in COVID-19 patients [4,11,12]. Various guidelines on the clinical management of COVID-19 patients in different countries were published and frequently revised, but discrepancies remain. For instance, the latest WHO guidelines in November 2021 state that antibiotics should not be prescribed for mild and moderate patients without a clinical suspicion of bacterial infection, but for severe patients, they recommend empirical antibiotic therapy to treat all likely pathogens [13]. The current UK National Institute for Health and Care Excellence (NICE) COVID-19 Rapid guideline: Managing COVID-19, however, clearly states that antibiotics for COVID-19 patients should be used with caution unless there is suspicion or confirmation of bacterial infection, and the guideline does not warrant the use of antibiotics for severely ill patients [14]. The U.S. National Institutes of Health (NIH), in turn, emphasize that in patients with severe or critical COVID-19, there is insufficient evidence for the panel to make an argument either for or against the use of empiric broad-spectrum antimicrobial therapy in the absence of another indication [15]. Updating the clinical guidelines, especially from authoritative sources, can reassure clinicians or increase their confidence in antibiotic prescribing in COVID-19 patients. For example, the UK NICE guidelines, on 3rd June 2021, advised against the use of Azithromycin for treating COVID-19 patients after weighing its potential side effects and implications for AMR against the limited benefits to patients’ treatment outcomes [14]. There is no doubt that antibiotic stewardship should be implemented when treating COVID-19 patients; however, which specific groups of COVID-19 patients should be given antibiotics, and when and how antibiotics should be administered, remain uncertain. Further research evidence on the scale of antibiotic use for COVID-19 patients in different economic settings and geographical regions; rationales for antibiotic prescribing in COVID-19 patients; and factors associated with empirical antibiotic therapy, the prevalence of bacterial infection and patterns of bacterial resistance in COVID-19 patients will contribute to a more accurate assessment of the scale of and rationales for antibiotic use for COVID-19 patients; moreover, it will contribute to the improvement of AMR surveillance, antibiotic stewardship programs, and infection prevention and control practices in future waves of the pandemic.

In this scoping review, we aimed to document the potential impact on AMR of antibiotic use in the treatment of COVID-19 patients by investigating:

1. Changes in the global scale and patterns of antibiotic use in COVID-19 patients since the early stages of the pandemic.
2. The proportion of antibiotic use based on the suspicion or confirmation of bacterial infection in COVID-19 patients and the proportion of antibiotic use as an empirical treatment.
3. The prevalence of bacterial infection in COVID-19 patients and rates of secondary bacterial infection and bacterial co-infection. These were defined as follows, with the duration of time since hospital admission acting as a proxy for defining hospital-acquired vs. community-acquired infection: Secondary infection—bacterial infection that developed during a hospital stay of >3 days; co-infection—bacterial infection that was already present at admission or detected within 3 days of hospital admission).
4. Frequently prescribed antibiotics and the most commonly resistant pathogens in COVID-19 patients.
5. The impact of antibiotic use on the clinical outcomes of COVID-19 patients.
6. The impacts of bacterial infection on the clinical outcomes of COVID-19 patients.

## 2. Materials and Methods

This scoping review was undertaken to identify, synthesize and analyze findings from studies that reported antibiotic use and bacterial infection in COVID-19 patients and were published in the second phase of the pandemic, from June 2020 to March 2021 (prior to the widespread rollout of vaccines); moreover, it aims to examine whether this has changed since the first phase of the pandemic from December 2019 to June 2020 [4]. This review is reported in line with Preferred Reporting of Systematic Reviews and Meta-Analyses (PRISMA) extension for Scoping Reviews (http://www.prisma-statement.org/Extensions/ScopingReviews, accessed on 1 April 2021) [16], and the protocol is registered with the Open Science Framework (OSF): http://osf.io/6zb3v, accessed on 1 July 2021.

### 2.1. Search Strategy

The following databases were searched to identify relevant studies from 15 June 2020–31 March 2021: Web of Science, EMBASE, PubMed and two Chinese academic databases (CNKI and VIP). No limits were set on the country in which studies were conducted, and we excluded any studies not available in English or Chinese. The search terms deployed were the same as those used in our previous scoping review on antimicrobial use in COVID-19 patients during the first phase of the pandemic [4].

### 2.2. Inclusion and Exclusion Criteria and Study Selection Process

Articles fulfilling the following criteria were considered for inclusion in the review. Only full-text articles were included:

#### 2.2.1. Inclusion Criteria

1. All types of clinical studies (cohort, cross-sectional, case–control, randomized control trials (RCT), case reports including case series, other observational studies and some qualitative studies including surveys) reporting the use of antibiotics to treat patients with COVID-19.
2. Studies reporting patients diagnosed with COVID-19 and receiving antibiotic treatment, without restrictions on age, race, gender, geographical location or setting.
3. Studies which reported both antibiotic treatment and clinical outcomes for COVID-19 patients.

#### 2.2.2. Exclusion Criteria

1. Animal studies, in vitro experiments, in silico screening/drug modeling, molecular mechanisms and other aspects of COVID-19 research that were not related to or did not mention antibiotic use (ABU).
2. Conference abstracts.
3. Commentaries and editorial notes and letters.
4. Perspectives.
5. Literature reviews.
6. Trial protocols.
7. Clinical updates or guidelines.
8. Case reports and case series with a documented sample size of less than 10 COVID-19 patients.
9. Full-text articles not available in English or Chinese.

Titles and abstracts were screened initially, and full texts were retrieved from articles which appeared to fulfil the inclusion criteria. Two independent, duplicate screenings were undertaken by WC, NA, BL and YT for all search results. Disagreements were resolved by consensus. Additional studies were identified by searching the reference lists of retrieved articles and the authors’ reference collections.

### 2.3. Data Extraction

A bespoke data-extraction form was modified from our published review [4]. Data extraction for antibiotic use in the treatment of COVID-19 patients included: publication details; country; region; World Bank classification for national economic status of study country (high, upper-middle, lower-middle, low) [17]; patient characteristics; sampling period; total number of patients reported; setting (inpatient or outpatient, including patients from emergency departments and care homes); COVID-19 patient category (severe or critical; mild or moderate); total number of patients prescribed antibiotics, and subtotals for mild or moderate and for severe or critical patients prescribed antibiotics, if reported; types of antibiotics prescribed; reasons for prescribing antibiotics; age; gender; mean length of hospital stay; discharge rate; and mortality rate, if reported.

Data extraction for studies reporting bacterial infection in COVID-19 included: publication details; country; region; national economic status of study; sampling period; total number of COVID-19 patients reported; total number of COVID-19 patients reported with a bacterial infection; percentage of patients with bacterial infection; total number of severely ill patients and number of severely and critically ill patients with bacterial infection; total number of patients with mild and moderate symptoms and number of mildly and moderately ill patients with bacterial infection; reported bacterial infection type (co-infection or secondary infection); any resistant pathogens, if reported; age; gender; mean length of hospital stay; discharge rate and mortality rate, if reported; underlying health conditions; and antibiotic prescribing details for patients with bacterial infection, if reported.

### 2.4. Data Synthesis and Analysis

In this review, data extraction and analysis were performed in Microsoft Excel. Descriptive synthesis was conducted, taking into account of the sample size of each study (i.e., weighted mean) while calculating pooled estimates of outcome variables (i.e., antibiotic prescribing rate, percentage of bacterial infection, mortality rate, discharge rate and length of stay). We also analyzed the most frequently used antibiotics for COVID-19 patients; summarized common antibiotic prescribing scenarios for COVID-19 patients; and identified the most frequently reported resistant pathogens in COVID-19 patients.

We conducted subgroup analyses on: (1) antibiotic prescribing rate, percentage of bacterial infection, health outcomes (length of stay, discharge rate and mortality rate) for patients with different severities of illness (mild and moderate, severely and critically ill); (2) health outcomes for patients with different antibiotic prescribing rates (100%, more than 50% and less than 50%); and (3) health outcomes for patients with bacterial infection.

In addition, we also conducted a stratified analysis of major outcome variables (antibiotic prescribing rate, length of hospital stays, discharge rate and mortality rate) by income economy of study country, region, and patient category and study type.

## 3. Results

### 3.1. Study Selection

**Figure 1:**
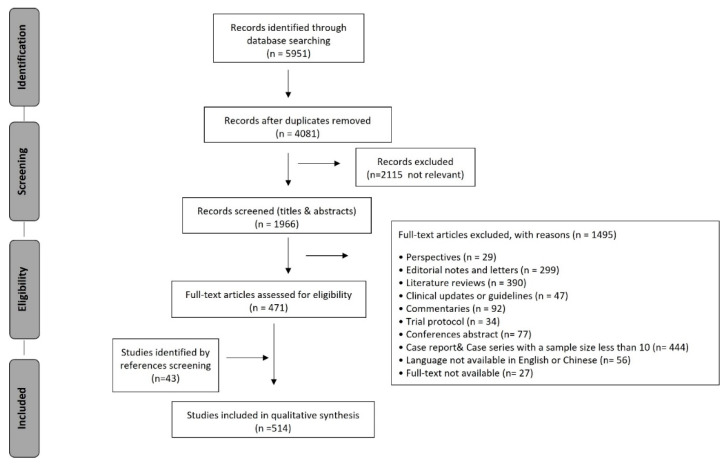
PRISMA Chart.

A total of 5951 records were identified through a database search. After the duplicates were removed, and through title and abstract screening, irrelevant records on COVID-19 research that were not related to the clinical treatment of COVID-19 patients or bacterial infection in COVID-19 patients were excluded. A total of 1966 records were screened for eligibility. A further 1495 records—including reviews; perspectives; editorial notes and letters; commentaries; conference abstracts; trial protocols and clinical guidelines; case reports and case series with a sample size of less than 10 COVID-19 patients; and records for which the full text was not available in English or Chinese—were excluded. The remaining 471 articles and an additional 43 articles, which were identified by searching the reference lists of the retrieved articles and the authors’ reference collections, led to a total of 514 full-text articles being included for review (Figure 1).

### 3.2. Description of Included Studies

Of the 514 included studies, 27 studies exclusively reported bacterial infection in COVID-19 patients and 69 studies reported both antibiotic use and bacterial infection in COVID-19 patients; the remaining 418 studies exclusively reported on antibiotic use in COVID-19 patients. This produced a total of 487 included studies on antibiotic use and 96 included studies on bacterial infection.

### 3.3. Antibiotic Prescribing for COVID-19 Patients

The antibiotic prescribing rate for COVID-19 patients was not reported in all 487 studies; only 433 studies with available antibiotic prescribing data were included for data synthesis. We found that 163,902 out of 412,476 COVID-19 patients were prescribed antibiotics and the average antibiotic prescribing rate was 39.7%.

#### 3.3.1. Antibiotic Prescribing and Healthcare Settings

Of the 433 studies available for antibiotic use data synthesis, 375 studies reported on inpatients from hospitals, 14 studies reported on outpatients, including patients from A&E departments and care homes, and 44 studies reported on both inpatients and outpatients, but without antibiotic prescribing rates based on healthcare settings. The overall antibiotic prescribing rate for hospitalized COVID-19 patients was 52.8% compared to 22.7% for COVID-19 patients without hospital admission (Table 1).

#### 3.3.2. Antibiotic Prescribing and Illness Severity

From a total of 182,887 COVID-19 patients reported in the included studies, 29,912 from 176 studies were identified as severe or critical and 152,975 from 144 studies were characterized as mild or moderate. The average prescribing rate for mild and moderate COVID-19 patients was 15.5%, compared to 48.3% for severe and critical COVID-19 patients (Table 2).

#### 3.3.3. Antibiotic Prescribing for COVID-19 Patients over the Course of the Pandemic

Compared with our review data of studies from the first phase of the pandemic from December 2019 to June 2020 [4], the average antibiotic prescribing rate for COVID-19 patients overall in the second phase, from June 2020 to March 2021, decreased from 82.3% to 39.7%; from 75.1% to 15.5% for mild and moderate COVID-19 patients; and from 75.4% to 48.3% for severe and critical COVID-19 patients (Table 3).

#### 3.3.4. Antibiotic Prescribing and Health Outcomes

We explored the relationship between antibiotic prescribing and health outcomes (length of hospital stay (LOS), discharge rate and mortality rate) and found that the mortality rate was higher in studies in which all patients were given antibiotics and studies in which most patients were given antibiotics, as compared to studies in which most patients were not given antibiotics (15.9% and 16.9% vs. 9.0%). The discharge rates (75.3% vs. 75.7%) and LOS (14.2 days vs. 14.5 days) were similar between the latter two groups of studies (Table 4).

#### 3.3.5. Severity Categories and Outcomes

As most studies only reported the overall clinical outcomes in COVID-19 patients, it was difficult to accurately assess clinical outcomes by severity of illness. A total of 47 studies reported exclusively on mild and moderate COVID-19 patients, and 86 studies reported the outcomes for severe and critical COVID-19 patients; we compared the clinical outcomes from these studies by severity. Severe and critical COVID-19 patients had a higher mortality rate (27.1% vs. 2.0%), lower discharge rate (59.8% vs. 79.2%) and longer LOS (15.6 vs. 12.0 days) than mildly and moderately ill patients (Table 5).

#### 3.3.6. Antibiotic Prescribing with Health Outcomes by Country Economic Status, Geographical Region, and Study Type

We conducted a stratified analysis of antibiotic prescribing with the health outcomes of COVID-19 patients by national economic status of the study country (Table 6), geographical region (Table 7) and study type (Supplementary Table S1). Interestingly, high-income economies had a higher mortality rate (14.3% vs. 9.1%), shorter LOS (13.7 vs. 15.3 days) and higher antibiotic prescribing rate (50.0% vs. 28.5%) compared to low- and middle-income economies.

A total of 206 studies were from East Asia and the Pacific (including 187 studies from China), Europe and Central Asia (134 studies) and North America (44 studies). South Asia had the highest antibiotic prescribing rate (88.7%), followed by the Middle East and North Africa (82.2%), and North America (73.7%); Europe and Central Asia had the highest mortality rate of 21.2%, and East Asia and the Pacific had the longest LOS (16.2 days) for COVID-19 patients.

### 3.4. Frequently Prescribed Antibiotics for COVID-19 Patients

**Figure 2:**
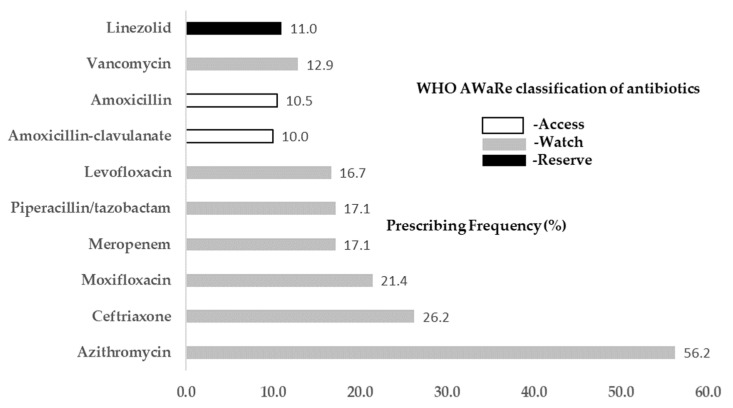
Frequently prescribed antibiotics in COVID-19 patients.

Azithromycin, ceftriaxone, moxifloxacin, meropenem and piperacillin/tazobactam remained the top five most frequently prescribed antibiotics for COVID-19 patients (Figure 2). The seven most frequently prescribed antibiotics (70%) are all on the WHO AWaRe [18] classification’s “Watch” list, while only two frequently prescribed antibiotics (20%; amoxicillin and amoxicillin–clavulanate), are in the “Access” category. Linezolid, which should be reserved as a last-resort treatment, was still frequently used for treating COVID-19 patients. Macrolides, cephalosporins (especially third- and fourth-generation cephalosporins) and fluoroquinolones (Supplementary Table S2) were other popular classes of antibiotics for treating COVID-19 patients.

### 3.5. Frequent Antibiotic Prescribing Scenarios for COVID-19 Patients

We used antibiotic prescribing scenarios (modified from our published review [4]) to classify each study that reported any rationale for prescribing, as shown in Table 8. Around 16% of the studies (77 studies) reported a microbiological analysis of bacterial infections when antibiotics were prescribed for COVID-19 patients; 54% of the studies (265 studies) were classified as having probably used antibiotics as an empirical treatment, among which 143 studies stated, explicitly, that antibiotics were used as an empirical/adjuvant/concomitant/standard treatment; and another 122 studies stated that antibiotics were used for treating COVID-19 patients without an explanation of the reasons, so we assumed that antibiotics were likely to have been used empirically/presumptively. Only 28% of the studies (76/265 studies) focused exclusively on specific COVID-19 patient groups: pregnant women; pediatric patients and elderly patients; patients with existing health conditions such as cancers, organ transplants, diabetes, obesity and hypertension; and patients with other kidney diseases, liver diseases, lung diseases and gastrointestinal diseases.

### 3.6. Bacterial Infection in COVID-19 Patients

A total of 3447 out of 32,751 COVID-19 patients from 82 studies were reported as having bacterial infections, and the average bacterial infection rate in COVID-19 patients was 10.5%. We categorized bacterial infection as either co-infection (community-acquired infection and patients who acquired the infection before or within 3 days of hospital admission) or secondary infection (patients who developed bacterial infection after a hospital stay of more than 3 days). We found secondary infection to be more common than co-infection (13.5% vs. 7.0%) in the included studies, suggesting the importance of hospital acquisition (Table 9).

We also investigated the severity of patients with bacterial infections in relation to the health outcomes of patients; around 20.3% of severe and critical patients developed bacterial infections (1583/7782 patients from 34 studies), whereas only 3.8% of mild and moderate patients were reported to have had bacterial infections (Table 10).

### 3.7. Frequently Reported Resistant Pathogens in COVID-19 Patients

**Figure 3:**
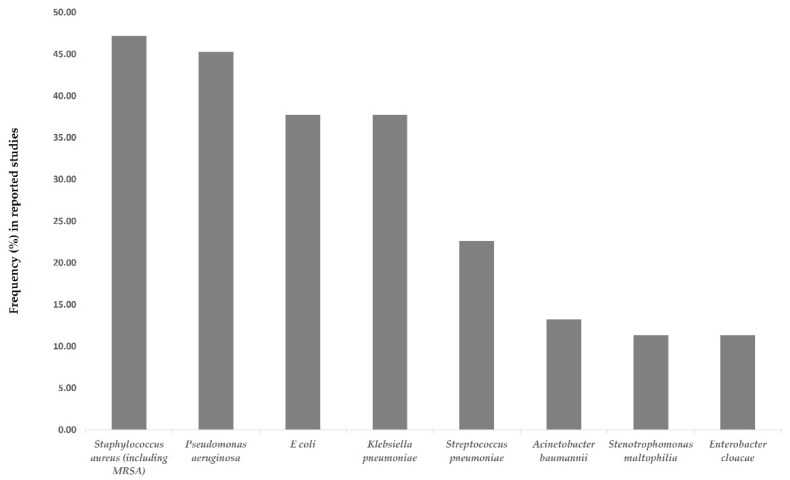
Frequently reported resistant pathogens in COVID-19 patients.

A total of 53 studies provided data on resistant pathogens for COVID-19 patients with bacterial infections. Bacterial resistance was most frequently reported for *Staphylococcus aureus* (25 studies), *Pseudomonas aeruginosa* (24 studies), *E. coli* (20 studies), *Klebsiella pneumoniae* (20 studies) and *Streptococcus pneumoniae* (12 studies) (Figure 3). Besides *Streptococcus pneumoniae* and *Coagulase negative staphylococci*, the other frequently reported pathogens are all Gram-negative.

## 4. Discussion

The widespread use of antibiotics for COVID-19 patients regardless of illness severity during the first wave of the pandemic, from December 2019 to June 2020 [4,8,9], has led to concern, about the potential consequences for the ‘slow pandemic’ of AMR [19]. The current review was designed to explore whether the prevalence of antibiotic prescribing for COVID-19 patients has changed since the start of the pandemic. Indeed, we found that the average antibiotic prescribing rate in COVID-19 patients decreased substantially from 82.3% in the first phase, from December 2019 to June 2020 (as reported in a previous review of studies published during this period), to 39.7% over the period covered by the current review, from June 2020 to March 2021; this is in keeping with observations from other studies [20,21,22]. Of particular note was a substantial reduction in antibiotic prescribing (from 75.4% to 15.5%) for mildly and moderately ill patients. Many reasons, including changes in government policies, clinical guidelines, individual preventive and healthcare-seeking behaviors, and increasing knowledge about the clinical management of the disease, may account for this. For instance, once the mortality risks for COVID-19 had been established, in most countries, COVID-19 patients with mild symptoms were recommended to self-manage the infection at home and to recover by taking sufficient rest, ensuring adequate energy intake, and taking symptom-relieving medicines which did not include antibiotics. This would substantially reduce COVID consultations and, hence, antibiotic prescribing for COVID-19 patients. As advances have been made in understanding the pathophysiology, transmission, diagnosis, treatment, and prevention of COVID, clinicians are likely to have become more confident in providing appropriate treatment for COVID-19 patients. Clinicians’ attitudes towards antibiotic prescribing in COVID-19 patients may have also been influenced by the introduction of treatment guidelines for COVID-19 patients (such as those issued by the WHO [13] and UK NICE [14]), which advised against antibiotic use in mild COVID-19 patients and in moderately ill patients without a suspected or confirmed bacterial infection. A cross-sectional study in Pakistan [23] that evaluated physicians’ perceptions, attitudes and confidence about AMR and antibiotic prescribing in COVID-19 patients from Apr to May 2021, found that most physicians had a high awareness of AMR during the pandemic and were confident about antibiotic prescribing in COVID-19 patients.

At the time of the studies reviewed, antibiotic use was still recommended for severe and critical COVID-19 patients to cover all likely pathogens, but we also found a considerable decline in antibiotic use for these patients over the review period compared to the initial phase of the pandemic (45.3% vs. 75.4%), as reported in our previous review [4]. This suggests that clinicians became increasingly cautious about prescribing antibiotics for COVID-19 patients in conjunction with the development of knowledge about appropriate treatment, and that antibiotic stewardship was, to some extent, re-established during subsequent phases of the pandemic.

However, our review shows that only 16% of the studies reporting antibiotic use for COVID-19 patients reported indications of bacterial infection, while around half (54%) of all the studies involved the use of antibiotics on an empirical basis. Only 28% of these were related to COVID-19 patients from vulnerable groups who might have been deemed at potentially higher risk from bacterial infection, suggesting that there continues to be substantial overuse of antibiotics in the treatment of COVID-19.

The WHO recommends a country-level target of at least 60% of total antibiotics consumed being from the “Access” list of antibiotics, according to the WHO AWaRe classification [18]. WHO guidelines recommend initial use of an antibiotic from the “Access” list when there is clinical suspicion of bacterial infection, followed by early discontinuation of antibiotic therapy if bacterial infection is not confirmed. However, we found that only 20% of frequently used antibiotics for COVID-19 patients belonged to this category, while 70% were on the “Watch” list. Similarly, a study from Sierra Leone reported that around 73% of prescribed antibiotics for COVID-19 patients were in the “Watch” category [24], and a study from Pakistan found that all the prescribed antibiotics for COVID-19 patients were either in the “Watch” or “Reserve” categories [25]. “Watch” antibiotics are at a relatively higher risk of selection for bacterial resistance, and most antibiotics on the “Watch” list are among the Critically Important Antimicrobials for Human Medicine [18]. The widespread use of “Watch” antibiotics will increase the risk of drug resistance in the pathogens they are used to treat; this renders these critical antibiotics ineffective, thus leading to more difficult-to-treat infections and more deaths. The widespread use of “Watch” antibiotics in COVID-19 patients suggests an urgent need for more widespread dissemination of information and clinical guidelines to inform clinicians’ choices for antibiotic prescribing in COVID patients, to support more prudent use of antibiotics and limit the further development of antibiotic resistance.

In June 2021, UK NICE [14] issued the recommendation not to use Azithromycin, which has been the most frequently prescribed antibiotic for COVID-19 throughout the pandemic, to treat COVID-19 patients. Several studies of azithromycin in the treatment of both seriously ill hospitalized COVID-19 patients and milder cases in community settings showed no meaningful benefit in any of the critical outcomes [26,27], while there are worrying risks of cardiotoxicity associated with macrolide antibiotics [28]. Although the WHO has not explicitly stated that it is against the use of azithromycin, it emphasizes that antibiotic prescribing for suspected or confirmed COVID-19 patients with low suspicion of a bacterial infection should be cautious, to avoid more short-term side effects of antibiotics in patients and negative long-term consequences of increased antimicrobial resistance [13]. This implies that authorities have started to evaluate the benefits/risks of using antibiotics in COVID-19 patients. Interestingly, in both this and our earlier review [4] we found a higher mortality rate and a similar discharge rate and length of stay in studies wherein the majority of patients were given antibiotics, compared to those in which the majority of patients were not given antibiotics. This suggests that antibiotic use may not be associated with improved outcomes for COVID-19 patients.

Comparison of data from published studies in the early stages (December 2019 to June 2020) and the following phase (June 2020 to March 2021) of the pandemic suggests that the bacterial infection rate for COVID-19 patients changed little over the period (from 9.5% 4 to 10.5%). This is consistent with findings from other publications [29,30,31]. Our finding of a higher incidence of secondary bacterial infection (13.5%) compared to co-infection (7%) in the reported studies suggests that COVID-19 patients are at higher risk of acquiring bacterial infection during their hospital stay than from community settings. Predictably, severe and critical COVID-19 patients who normally need longer hospital stays and are more likely to receive ventilation support—for example, up to 80% of the COVID-19 patients admitted to the ICU required invasive mechanical ventilation [32,33]—have a higher prevalence of nosocomial pneumonia, especially ventilator-associated pneumonia [33]. Commensurate with this, our analysis found that severe and critical COVID-19 patients are at higher risk of acquiring a bacterial infection (20.3%) than mild and moderate COVID-19 patients (3.8%). We also found, in both our previous [4] and the current review, that COVID-19 patients with bacterial infection (whether secondary or co-infection) tend to have long hospital stays (24.6 days) and high mortality rates (30.3%).

It is difficult to infer that bacterial infection might be associated with increased COVID-19 mortality; this is because it is not possible to determine whether COVID-19 patients with a bacterial infection who did not survive started to decline before or after the onset of bacterial infection [34,35]. Bacterial infection might only be an indicator rather than a cause of an overall decline in health conditions, so the relationship between bacterial infection and COVID-19 mortality remains unclear. More experimental laboratory models of bacterial infection with SARS-CoV-2 would be required to enable a well-controlled and comprehensive investigation of the differences in the molecular mechanisms between viral and bacterial dynamics and host outcomes, in order to elucidate the underlying mechanism and impacts of bacterial infection on COVID-19 mortality.

*Staphylococcus aureus*, including methicillin-resistant *S. aureus* (MRSA), was the most frequently reported resistant pathogen in COVID-19 patients in our included studies, as further evidenced in other publications [36,37]. As Staphylococcus aureus can potentially cause various respiratory complications such as necrotizing pneumonia or pneumothorax [38], and people with MRSA are 64% more likely to die than people with drug-sensitive infections [39], it is very important to establish appropriate clinical management and treatment regimens for hospitalized COVID-19 patients with suspected or confirmed *S. aureus* infection. UK NICE recommends vancomycin or teicoplanin, or linezolid if vancomycin cannot be used, and dual therapy with another first-choice intravenous antibiotic for treating suspected or confirmed MRSA infection [40]. However, vancomycin is gradually losing its efficacy as MRSA strains are developing resistance against it [41]. Therefore, the therapeutic alternatives available for treating MRSA infection are actually very limited, and there is a global urgency for the development of novel antibiotics for treating MRSA and *S. aureus* infections. Resistance was already globally widespread for the other frequently reported pathogens such as *Pseudomonas aeruginosa*, *E. coli* and *K. pneumoniae* before the pandemic [42], especially in lower-income countries; for example, in 2017, the resistance rate to ceftriaxone in *E. coli* was 85% in Pakistan, 11.2% in Australia, and 11% in the UK. Given that ceftriaxone was the second most frequently used antibiotic in COVID-19 patients throughout the pandemic, resistance to ceftriaxone in *E. coli* is only likely to have been augmented during the pandemic. This suggests that addressing the challenges of AMR in the COVID crisis remains a high priority that cannot be delayed.

This large review of 514 studies published during the second phase of the pandemic, from June 2020 to March 2021, with a coverage of 412,476 COVID-19 patients from 35 countries around the world, has provided the most up-to-date synthesis of the available data on the reported prevalence and patterns of antibiotic prescribing and bacterial infection for COVID-19 patients after the early stage of the pandemic. Inevitably, this review has several limitations. First, we did not assess the quality of the included studies or conduct a meta-analysis due to the heterogeneity of the studies. We included all study types such as cohort studies, cross sectional studies, randomized control trials, case–control studies, case series with a sample size of >10 COVID-19 patients, surveys, and other observational studies in this review; we also did not set any limits on patient characteristics, so patients from specific groups (elderly patients, pregnant women, pediatric patients and patients with any existing health conditions) whose antibiotic prescribing may differ from the general population were also included. Furthermore, there were major variations in sample size among the included studies ranging from 10 to 136,855 COVID-19 patients. As most of the included studies were descriptive in nature, we could only manage a simple descriptive analysis of the information available and were unable to perform significance testing. Secondly, due to there being very few published studies from low- and middle-income countries (LMICs), except China, our findings have limited generalizability and may not capture the full picture of antibiotic prescribing patterns and prevalence in LMIC settings. As easy access to antibiotics without prescription, poorly resourced health facilities, limited AMR surveillance and a lack of enforcement of legislative regulations to control unnecessary or inappropriate antibiotic use are characteristic of many such settings [43,44,45], antibiotic use for COVID-19 in LMICs is likely to be substantially underrepresented in this review. A systematic review and meta-analysis to investigate the prevalence and patterns of antibiotic prescribing and bacterial infection for COVID-19 patients in LMICs is underway. Third, this study was an evidence synthesis of aggregated data from studies published between June 2020 and March 2021. We mainly focused on examining the prevalence and patterns of antibiotic prescribing and bacterial infection in COVID-19 patients during this period, so causal conclusions regarding the relationships between antibiotic prescribing, bacterial infection and health outcomes could not be established. Fourth, although we compared the prevalence and scales of antibiotic prescribing and bacterial infection in COVID-19 patients based on our previous review of studies published in the first phase of the pandemic (from December 2019 to June 2020) [4] and the second phase (from June 2020 to March 2021) covered by the current review, the studies included in each phase did not necessarily involve the same localities or patients; thus, these comparisons over time need to be interpretated cautiously, particularly given the emergence of new coronavirus variants in later stages of the pandemic.

## 5. Conclusions

The first phase of the pandemic (from December 2019 to June 2020) was associated with widespread and indiscriminate antibiotic prescribing for COVID patients, which rapidly reduced in the second phase of the pandemic from June 2020 to March 2021. Although there are many possible reasons for this decline, it does suggest that clinicians became increasingly cautious about prescribing antibiotics to treat COVID-19 patients, and antibiotic stewardship seems to have been re-established to some extent. Nonetheless, we also found evidence for extensive inappropriate use of “Watch” antibiotics for COVID-19 patients that is cause for continued concern. Since the widespread resistance of common pathogens such as *E. coli* and *K. pneumoniae* to several of the antibiotics that have been frequently prescribed for COVID-19 patients was already established before the pandemic, the continued unwarranted use of these antibiotics in the absence of any evidence of clinical benefit is liable to augment antibiotic resistance. There is an urgent need to establish comprehensive and consistent guidelines to assist clinicians in selecting appropriate antibiotics for COVID-19 patients in the limited circumstances in which these are needed; moreover, sensitivity data for antibiotics frequently used in COVID-19 patients for prevalent pathogens should also be closely monitored. These measures may help to reduce unnecessary antibiotic use in COVID-19 patients and to monitor the risk of AMR during the pandemic. AMR affects all countries, but the burden of AMR is disproportionately higher in LMIC settings, while antibiotic use for COVID-19 in most LMICs is underrepresented in this review. A further systematic review and meta-analysis to investigate antibiotic prescribing and bacterial infections for COVID patients in LMICs is underway.

COVID-19 patients with bacterial infection tend to require longer hospital stays and have higher mortality rates; the relationship between bacterial infection and COVID-19 mortality is still unknown, and robust clinical and epidemiological research will be required to elucidate the underlying mechanisms of bacterial infection associated with COVID-19.

## Figures and Tables

**Table 1:** Healthcare settings and antibiotic prescribing in COVID-19 patients from studies published between June 2020 and March 2021.

Patient Category	Number of Studies	Number of Prescribed Antibiotics	Total Number of Patients	Proportion of Prescribed Antibiotics
Inpatients	375	82,779	156,716	52.8%
Outpatients	14	8493	37,326	22.7%
Mixed (inpatients and outpatients)	44	72,630	218,434	33.3%

**Table 2:** Severity of illness and antibiotic prescribing in COVID-19 patients.

Illness Severity of COVID-19 Patients	Number of Studies	Number of Prescribed Antibiotics	Total Number of Patients	Proportion of Prescribed Antibiotics
Severe and critical	176	14,444	29,912	48.3%
Mild and moderate	144	23,761	152,975	15.5%

**Table 3:** Comparison of antibiotic prescribing rates by severity of COVID-19 illness in COVID-19 patients over the time of the pandemic.

Illness Severity of COVID-19 Patients	Average Antibiotic Prescribing Rate (%)	
	The First Phase of the Pandemic (from December 2019 to June 2020)	The Second Phase of the Pandemic (from June 2020 to March 2021)
Severe and critical	75.4%	48.3%
Mild and moderate	75.1%	15.5%
Overall	82.3%	39.7%

**Table 4:** Antibiotic prescribing categories and health outcomes in COVID-19 patients.

Category of Antibiotic Prescribing	LOS (Mean Days)	Discharge (Mean %)	Mortality (Mean %)
All given abs	16.4 (25 studies)	71.0% (40 studies)	15.9% (55 studies)
Majority are given abs	14.2 (102 studies)	75.3% (143 studies)	16.9% (208 studies)
Majority not given abs	14.5 (43 studies)	75.7% (68 studies)	9.0% (101 studies)

**Table 5:** Severity categories and health outcomes in COVID-19 patients.

Illness Severity of COVID-19 Patients	LOS (Mean Days)	Discharge (Mean %)	Mortality (Mean %)
Severe and critical (86 studies)	15.6 (10 studies)	59.8% (40 studies)	27.1% (68 studies)
Mild and moderate (47 studies)	12.0 (30 studies)	79.2% (25 studies)	2.0% (35 studies)

**Table 6:** Study country by national economic status, antibiotic prescribing and health outcomes in COVID-19 patients.

World Bank Classification	Antibiotic Prescribing Rate (%)	LOS (Mean Days)	Discharge (Mean %)	Mortality (Mean %)
High Income	50.0% (192 studies)	13.7 (86 studies)	72.2% (97 studies)	14.3% (177 studies)
Low and Middle income	28.5% (240 studies)	15.3 (91 studies)	79.1% (175 studies)	9.1% (219 studies)
Mixed	52.9% (7 studies)	9.3 (2 studies)	64.9% (3 studies)	13.0% (5 studies)

**Table 7:** Geographical region, antibiotic prescribing and health outcomes in COVID-19 patients.

Geographical Region	Antibiotic Prescribing Percentage	LOS (Mean Days)	Discharge (Mean %)	Mortality (Mean %)
East Asia and Pacific	53.7% (206 studies)	16.2 (80 studies)	80.2% (147 studies)	8.1% (186 studies)
Europe and Central Asia	34.7% (134 studies)	13.5 (57 studies)	72.3% (64 studies)	21.2% (123 studies)
Latin America and the Caribbean	14.8% (15 studies)	11.1 (10 studies)	73.8% (12 studies)	8.9% (17 studies)
Middle East and North Africa	82.2% (25 studies)	11.7 (16 studies)	61.4% (20 studies)	16.0% (22 studies)
North America	73.7% (44 studies)	13.5 (22 studies)	75.4% (26 studies)	11.6% (41 studies)
South Asia	88.7% (7 studies)	4.2 (1 study)	95.7% (3 studies)	10.0% (6 studies)
Mixed	53.5% (8 studies)	13.5 (3 studies)	64.9% (3 studies)	14.4% (6 studies)

**Table 8:** Frequent antibiotic prescribing scenarios in COVID-19 patients in the selected studies.

Antibiotic Prescribing Scenario	Reason for Antibiotic Prescribing for COVID-19 Patients, If Reported	Number of Studies Reported	% of Total Studies
Suspicious or confirmed bacterial infection	Microbiological analysis of samples such as blood, stool, urine or sputum culture was conducted	77	15.8%
Empirical antibiotic therapy *	Antibiotics were used as an empirical/adjuvant/concomitant/standard treatment	143	29.4%
	Antibiotics were prescribed, but unclear whether it was based on suspicion or confirmation of bacterial infections (likely empirical treatment)	122	25.1%

* 76 studies out of 265 studies (28%) using empirical antibiotic therapy were for pregnant women; pediatric patients; elderly patients; patients with existing health conditions such as cancers, organ transplants, diabetes, obesity and hypertension; and patients with kidney diseases, liver diseases, lung diseases, gastrointestinal diseases, etc.

**Table 9:** Bacterial infection type and infection rate in COVID-19 patients.

Bacterial Infection Category	Number of Studies	Number With Bacterial Infection	Total Number of Patients Accounting for Bacterial Infection	Bacterial Infection Rate (%)
Secondary infection	32	1954	14,416	13.5%
Co-infection	28	992	14,416	7.0%
Secondary infection and co-infection	6	198	1697	11.7%

**Table 10:** Severity of illness, bacterial infection rate and health outcomes in COVID-19 patients.

Description	Percentage of Severely/Critically Ill Patients (*n*)	Percentage of Mildly/Moderately Ill Patients (*n*)	LOS (Mean Days)	Discharge (Mean %)	Mortality (Mean %)
Patients with bacterial infection	22.7% (34 studies)	3.7% (34 studies)	21.8 (17 studies)	59.6% (19 studies)	30.2% (42 studies)

## Data Availability

The data are contained within the article or Supplementary Material.

## Supplementary Materials

The following supporting information can be downloaded at: https://www.mdpi.com/article/10.3390/antibiotics11080991/s1, Table S1: Study type and health outcomes; Table S2: Information on prescribed antibiotics in the selected studies.
