# Supplementary material for: Antibiotic Use and Bacterial Infection in COVID-19 Patients in the Second Phase of the SARS-CoV-2 Pandemic: A Scoping Review
Source: Antibiotics (Basel). 2022 Jul 23;11(8):991. doi: 10.3390/antibiotics11080991 (PMC9331316; doi:10.3390/antibiotics11080991)
Supplement: Supplementary file 1 [file antibiotics-11-00991-s001.zip › antibiotics-1822400-supplementary.pdf]

Supplementary Table S1. Study type and health outcomes in COVID-19 patients

Geographical region	Antibiotic prescribing percentage	LOS (Mean Days)	Discharge (Mean%)	Mortality (Mean%)
Case control	34.4% (6 studies)	10.5 (2 studies)	89.0% (4 studies)	16.2% (5 studies)
Case report/series	62.0% (26 studies)	12.8 (10 studies)	68.3% (18 studies)	17.0% (21 studies)
Cohort	35.5% (96 studies)	14.1 (47 studies)	75.8% (56 studies)	10.3% (95 studies)
Cross sectional	56.4% (5 studies)	11.8 (4 studies)	94.7% (5 studies)	6.5% (5 studies)
Qualitative	21.4% (6 studies)	10.0 (1 study)	80.5% (2 studies)	17.0% (4 studies)
Survey	44.9% (7 studies)	14.9 (3 studies)	100% (1 study)	0% (2 studies)
Other observational	56.0% (274 studies)	14.9 (120 studies)	75.8% (182 studies)	13.3% (254 studies)
RCT	50.9% (17 studies)	6.5 (2 studies)	69.9% (7 studies)	19.5% (14 studies)

Supplementary Table S2. Information on class of antibiotics frequently prescribed for COVID-19 patients in the selected studies

Class of antibiotic	Number of studies reported
Macrolides	154
Cephalosporins	154
Fluroquinones	104
Beta lactams	74
Carbapenems	68
Glycopeptides	43
Penicillins	42
Tetracyclines	23
